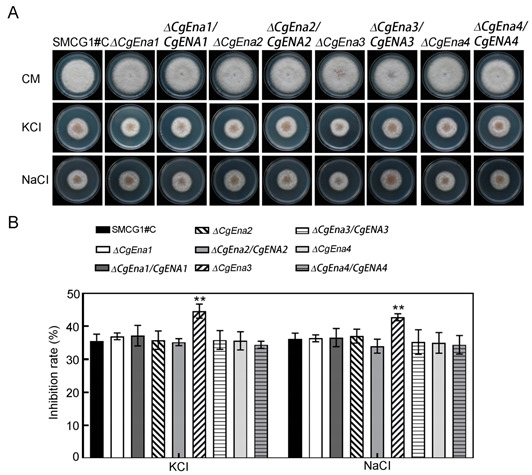# Correction: Deng et al. Distinct Roles of Ena ATP Family Proteins in Sodium Accumulation, Invasive Growth, and Full Virulence in *Colletotrichum gloeosporioides*. *J. Fungi* 2023, *9*, 566

**DOI:** 10.3390/jof9070743

**Published:** 2023-07-13

**Authors:** Tian-Ci Deng, Ji-Yun Yang, Mei-Ling Sun, Yun-Zhao Zhang, Yun-Ting Pan, Lin Huang

**Affiliations:** 1 Co-Innovation Center for Sustainable Forestry in Southern China, Nanjing Forestry University, Nanjing 210037, China; dtc919421@163.com (T.-C.D.); yangjiyun2018@163.com (J.-Y.Y.); sunmeiling0426@163.com (M.-L.S.); 15751455333@163.com (Y.-Z.Z.); panyuting1997@gmail.com (Y.-T.P.); 2 College of Forestry, Nanjing Forestry University, Nanjing 210037, China

## Error in Figure 3

In the original publication [1], there was a mistake in the published Figure 3. Because of our oversight during figure editing, we used similar images for the plate of Δ*Cgena3/CgENA3* and that of Δ*Cgena4/CgENA4* on CM medium supplied with 0.6 M KCl. The corrected Figure 3 appears below. The authors state that the scientific conclusions are unaffected. This correction was approved by the Academic Editor. The original publication has also been updated.