# Supplementary material for: Distinct Roles of Ena ATP Family Proteins in Sodium Accumulation, Invasive Growth, and Full Virulence in Colletotrichum gloeosporioides
Source: J Fungi (Basel). 2023 May 13;9(5):566. doi: 10.3390/jof9050566 (PMC10219246; doi:10.3390/jof9050566)
Supplement: Supplementary file 1 [file jof-09-00566-s001.zip › jof-2233290-supplementary.pdf]

Figure S1: Protein structure of the CgEna1/2/3/4 with its homologs in other fungi;

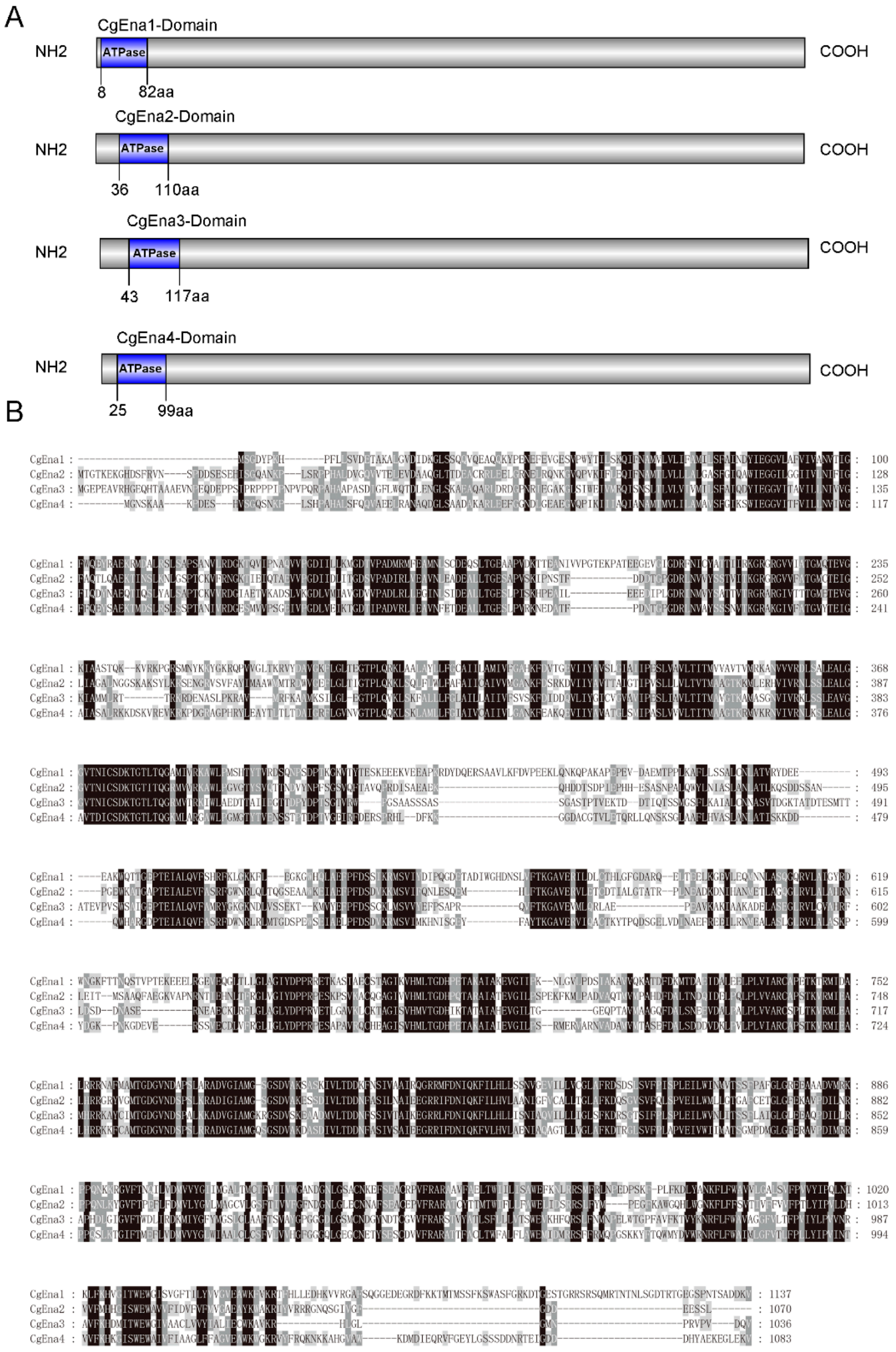

Figure S2: The expression of CgEna1/2/3/4 in mycelium and different stages of infection;

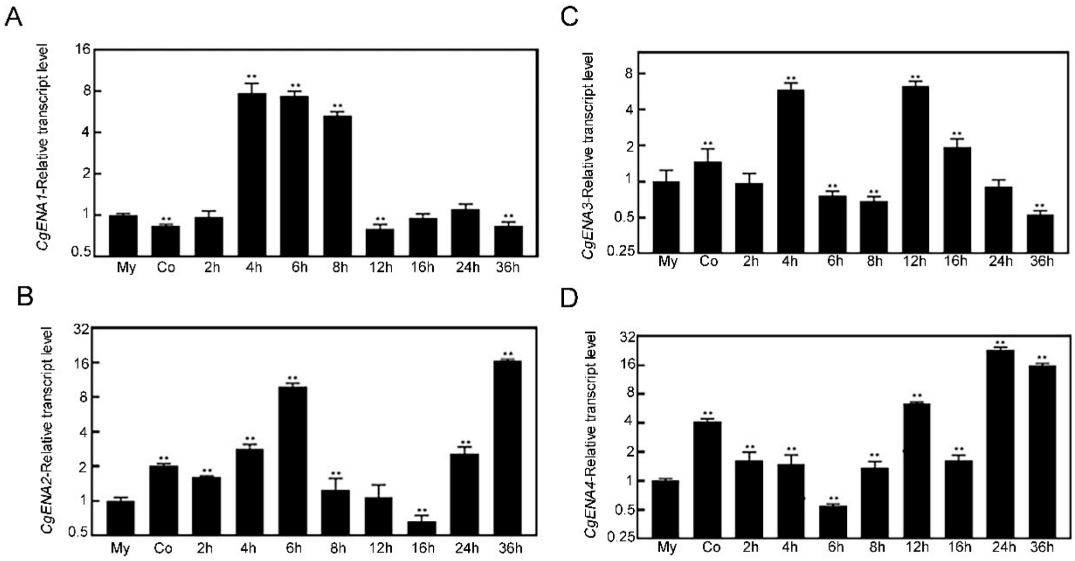

Figure S3: Acquisition and verification of mutants;

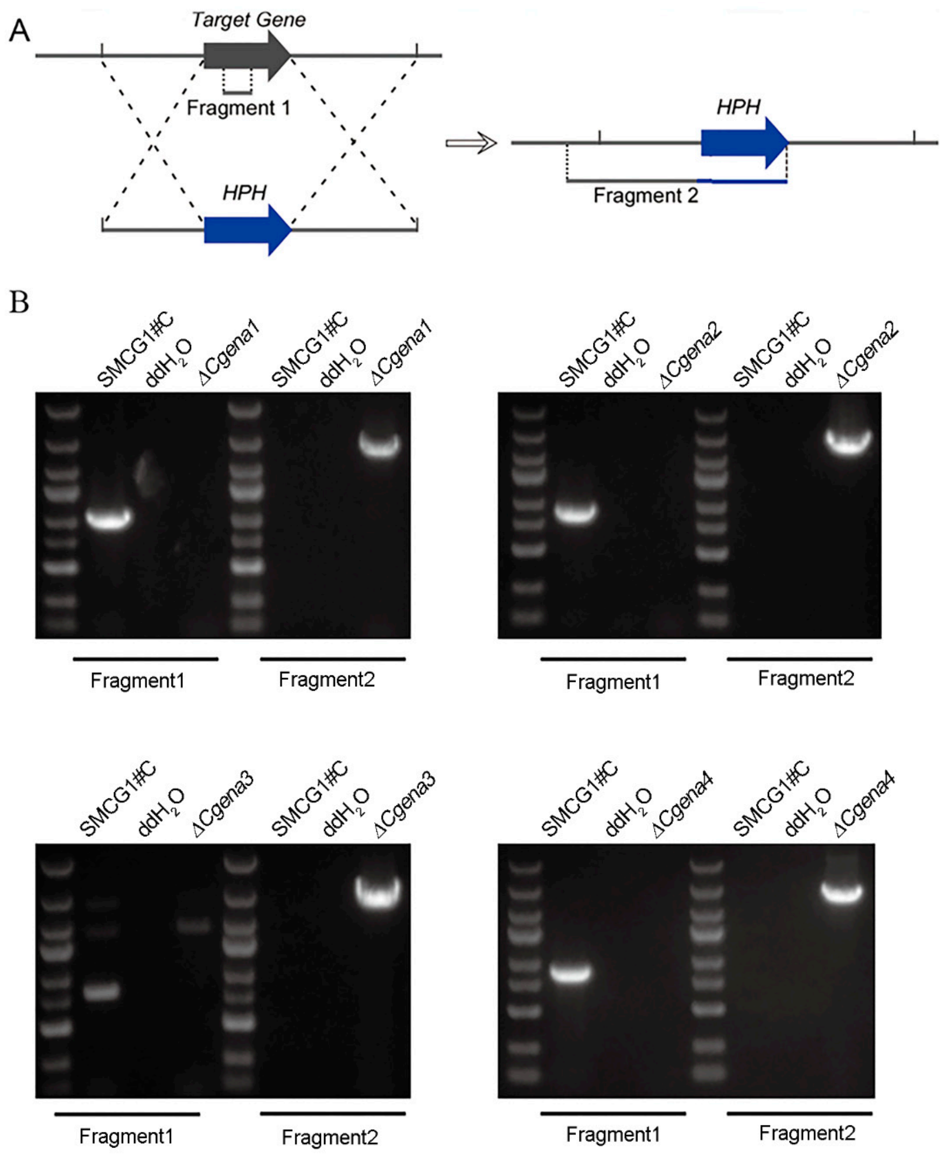

Figure S4: CgEna family proteins are not involved in the regulation of vegetative growth;

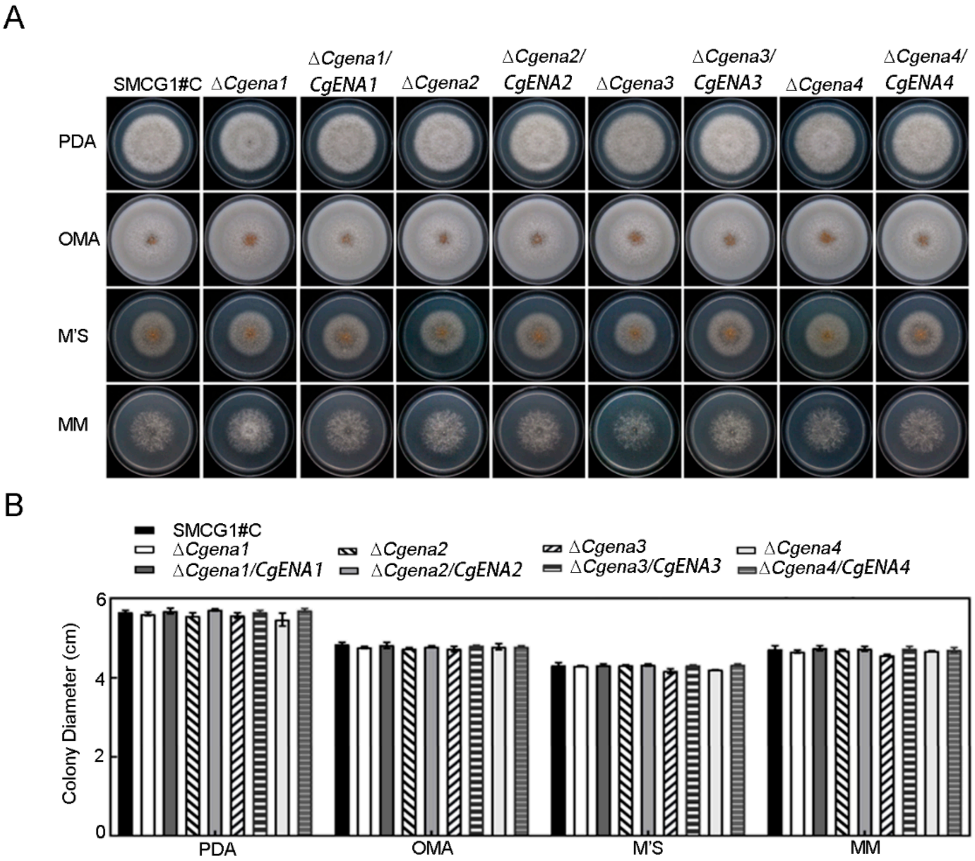

Table S1. Primer sequence of CgEna1/2/3/4.

Primer	Sequence (5’-3’)	Application
CgEna1-1F	CGAAGTCGGCGAGTCGGTGCC	Amplify *CgEna1* 5’ flank sequence, for gene knock out
CgEna1-2R	CGACAAGGTTTGATATCACGTGTACG	
HPH-1F	GGCTTGGCTGGAGCTAGTGGAGGTCAA	Amplify *HPH* sequence, for gene knock out
HPH-2R	CGGTCGGCATCTACTCTATTCCTTTG	
CgEna1-3F	CATTGGCGATCGCTTCAACATC	Amplify *CgEna1* 3’ flank sequence, for gene knock out
CgEna1-4R	AACACTGAGTTTGACACCG	
CgEna1-InnerF	ATGTCTGGTGACTACCCGAAGCACCCCTTCC	For *CgEna1* complementation
CgEna1-InnerR	TCAGACCTTGTCGTCCGCGCTGGTGTT	
CgEna1-OuterF	GACACGCCTCGCTGAACTCC	
CgEna1-GFP-1F	TTTCGTAGGAACCCAATCTTCAAAATGTCTGGTGACTACCCGAAGCACCCCTTCC	For *CgEna1*-GFPfusion construct, native promoter
CgEna1-GFP-2R	CACCACCCCGGTGAACAGCTCCTCGCCCTTGCTCACTCAGACCTTGTCGTCCGCGCTGGTGTT	
CgActin-1F	GTGAGGCCCTCAAAGGTAGTGG	Reference gene for qRT-PCR analysis
CgActin-2R	GGATCCCAGTGCGAGACGT	
CgEna1.qPCR.1F	CCAAGATTGTGCTCACTGACGA	*CgEna1 qRT-PCR* analysis
CgEna1.qPCR.2R	TCGAACATGCGACGACCCTG	
CgEna2-1F	TGCGCGAGTGACGAGGCG	Amplify *CgEna2* 5’ flank sequence, for gene knock out
CgEna2-2R	TGGAAACGAACTCATGG	
CgEna2-3F	TCGGCCGCAACGAGCTTCG	Amplify *CgEna2* 3’ flank sequence, for

CgEna2-4R	TCCCATAATAAACATCGCT	gene knock out
CgEna2-InnerF	ATGACTGGCACCAAGGAGAAA	For *CgEna2* complementation
CgEna2-InnerR	CTATAGAGATGATTCCTCATC	
CgEna2-OuterF	TGTCGGACCTGCTTGCG	
CgEna2-GFP-1F	TTTCGTAGGAACCCAATCTTCAAAATGACTGGCACCAAGGAGAAA	For *CgEna2* complementation
CgEna2-GFP-2R	CACCACCCCGGTGAACAGCTCCTCGCCCTTGCTCACCTATAGAGATGATTCCTCATC	
CgEna2.qPCR.1F	TTTATCCTCCACGTTCTAGCAG	*CgEna2 qRT-PCR* analysis
CgEna2.qPCR.2R	CTGATCTTTGAAGGCCAACCC	
CgEna3-1F	ACTGGCGTGGTGCTTG	Amplify *CgEna3* 5’ flank sequence, for gene knock out
CgEna3-2R	AGGAACGAGGAAGTAATGA	
CgEna3-3F	GCGCATGTTCTTGGCAAC	Amplify *CgEna3* 3’ flank sequence, for gene knock out
CgEna3-4R	TCTTCATAGCAGCCTTGAAG	
CgEna3-InnerF	ATGGGAGAACCCGAAGCCGTCA	For *CgEna3* complementation
CgEna3-InnerR	CTAGACCTGATCGACAGGAA	
CgEna3-OuterF	GCGCATGTTCTTGGCAAC	
CgEna3-GFP-1F	TTTCGTAGGAACCCAATCTTCAAAATGGGAGAACCCGAAGCCGTCA	For *CgEna3*-GFPfusion construct, native promoter
CgEna3-GFP-2R	CACCACCCCGGTGAACAGCTCCTCGCCCTTGCTCACCTAGACCTGATCGACAGGAA	
CgEna3.qPCR.1F	GGCCGTTATTCCCGAATCCCT	*CgEna3 qRT-PCR* analysis
CgEna3.qPCR.2R	ACACCTCCAACGGCTTCGAG	

CgEna4-1F	CGTGAGGTGCTGTGCTAT	Amplify *CgEna4* 5’ flank sequence, for gene knock out
CgEna4-2R	TTGTCTTGTCGAGTGCAGGCTG	
CgEna4-3F	CGAAGTTGGAATCCTGCCGTCCC	Amplify *CgEna4*3’ flank sequence, for gene knock out
CgEna4-4R	TCGTCGCCCACGTCCAGCC	
CgEna4-InnerF	ATGGGCAACTCAAAGGCAGC	For *CgEna4* complementation
CgEna4-InnerR	CTAAACCTTCTCCAAGCCTT	
CgEna4-OuterF	GGTAAGGTAAAGTACCTG	
CgEna4-GFP-1F	TTTCGTAGGAACCCAATCTTCAAA	For *CgEna4*-GFPfusion construct, native promoter
CgEna4-GFP-2R	CACCACCCCGGTGAACAGCTCCTCGCCCTTGCTCAC	
CgEna4.qPCR.1F	CTCGCTAGTCTTGGTCTTCGT	*CgEna4 qRT-PCR* analysis
CgEna4.qPCR.2R	GTCGTACAGGCCGATTAACCC	
